# Impact Study on Social Accounting Matrix by Intrabusiness Analysis

**DOI:** 10.3390/ijerph182312547

**Published:** 2021-11-28

**Authors:** Monica Laura Zlati, Romeo-Victor Ionescu, Valentin Marian Antohi

**Affiliations:** 1 Department of Accounting, Audit and Finance, Stefan cel Mare University, 720229 Suceava, Romania; sorici.monica@usm.ro; 2 Department of Administrative Sciences and Regional Studies, Dunarea de Jos University, 800201 Galati, Romania; ionescu_v_romeo@yahoo.com; 3 Department of Business Administration, Dunarea de Jos University, 800001 Galati, Romania; 4 Department of Finance, Accounting and Economic Theory, Transylvania University of Brasov, 500036 Brasov, Romania

**Keywords:** social corporative responsibility, social accounting matrix, business environment, intrabusiness transactions, environment impact

## Abstract

According to the current concerns about social welfare and environmental protection, integrated in a model assimilated to intrabusiness relations, our research started from the analysis of the initial model SAM, which will be transformed in order to develop the SAMI model under six research objectives. The need of improving SAM matrix started to connect it directly to the regional economic systems and continued to a new approach on Input-Output Analysis. Nowadays, SAM describes the intraregional connections between regional economic actors using the role of different income categories. Moreover, SAM can quantify different regional multipliers. All deficiencies previously identified in connection to SAM model have been reviewed and resolved within the proposed SAMI model by the authors of this paper. The purpose of this research is the launch of an absolutely new mathematical model (SAMI) and its practical testing at regional level. This model is able to systematize the links between the local and regional businesses, under the matrix (SAMI) flow, for all kinds of companies and to assist the regional decision, as well. Czamanski was not able to escape from the input-output prison’s approach. This is why he continued to use the linear interdependencies between the industries, economic sectors and economic actors. The income is able only to approximate the individuals and other economic actors’ welfare. If the increase in the average and aggregate income is doubled by an unfair distribution of income in two countries which have the same average income, the effects on welfare vary a lot. A relatively similar effect comes from the government policy differences in income distribution and redistribution.

## 1. Introduction

Regional accounting (regional accounts) was developed and put into connection to the regional development at the beginning of the ‘70s [1]. The model was well correlated to the input-output analysis and brought special matrix SAM (social accounting matrix), as it was defined by P. Batey and A. Rose [2].

Starting with this classic model, many pressures on the accounting system’s entropy have been identified, even that it was initially built in order to satisfy the needs of internal information users [3,4]. The process developed at the same time as the globalization’s increasing across the world economy.

The micro and macroeconomic diversification of the information actors and users, including EU27 and other regional organisations which developed common economic development systems, led to the entropy disturbing and pointed out the initial accounting system vulnerability in its relation to the new reporting needs. These needs were met by increasing external pressures on the system, along with external reporting regulations to which the accounting systems had to align.

Through his application, the inventor of the SAM matrix proved a visionary spirit that anticipated these entropy disturbances at the time of designing the model. The further evolution of the information exchange could not be predicted at the real level of its expansion. 

The SAM matrix is current because it is based in prediction on a consistent data system which captures socio-economic interdependencies and translates them into an input-output picture which defines the socio-economic framework of interest at a given time at regional or national level. The main drawback is the yearly fluctuations of the data, which reduces the quality of the forecast. Through the punctual nature of the analysis, the method allows the development of relevant policies based on interlinked observations from several areas of economic and social interest. This is the main advantage of the method and the reason why it is current, with respect to the criteria of objective data evaluation.

The SAM approach has led to enough updates, not infrequently contradictory. The basic problem is that the model (matrix) has to operate with a multitude of information coming from different sources, including data from prior years. This introduces the concept of flexible ‘cross entropy’ in the definition of SAM, which allows the introduction of the errors in variables and inequality constraints [5].

Starting from these considerations, the authors of this paper have proposed to reposition the social accounting matrix in relation to the new market demands, using the objective evaluation of the previous critical proposals on this instrument and developing a new concept by incorporating a very visible component: intrabusiness transactions, which reflect globally the amplitude of the international economy.

The main aim of this research is to improve the SAM matrix approach in direct connection with the regional dimension in the context of a new approach to the input-output theory. As a result, the objectives of the study were to: identify vulnerabilities for model SAM and intrabusiness sectoral flows, defining a social accounting matrix model based on intrabusiness exchanges, testing our proposed model, disseminating test results and formulating proposals for public policies’ decision makers.

The new model proposed is valid and applicable to any international region with conclusive results regarding the connections between the economic environment and the macro-environment through public policies in different sectors of activity.

In order to accurately and fluently identify the impact of inter-business transactions within the SAM matrix, the influences of technological exchanges have been marginalized, with the impact in particular on the dynamics of financial flows, which are currently the engine of the economy. These flows have faced a regress since Czamanski’s conceptualization of the model to date, based on free access to information and the digitization of the economy.

The paper is structured into five sections, of which the Introductory section studies the impact of the SAM concept on the current regional reality and the ways to improve it based on the need for IT-supported decision making. Section 2 contains the literature review addressing the dynamics of the concept and the main shortcomings that can be improved in the authors’ research. Section 3 presents the working methodology and design of the proposed new SAMI model, while Section 4 presents the main results of the research and discussions based on them. The last section Conclusions and policy implications presents the conclusions that the authors publicly disseminate with the research.

## 2. Literature Review

The social accounting represents an interesting subject for many specialists who responded to the challenge of updating the model.

In real economy, using their macroeconomic function, the companies make their contribution to the macroeconomic sector directly through the financial component (payment of taxes and fees) [6] and indirectly, through the social component (labour absorption, reduction in social disparities, regulation of the trade mechanism, etc.) [7,8].

The social accounting implies addressing cost-benefit measures on environmental issues [9], in order to limit pollution and other negative effects on the environment (reduction in biodiversity), but also complementary measures to boost the main macroeconomic indicators through the business component (GDP, total investment, reducing inflation by limiting deviations from the consumer price index [7,10].

There are social accounting studies which point out that the distributional structure of policies to mitigate disparities in social matters can be adjusted by effective measures to increase sectoral competitiveness and cost-benefit adjustment [11].

The intra-sectoral cooperation based on competitive exchanges linked to controlled excise limitation may increase the favorable impact of the competitiveness of domestic firms, including on foreign markets [12].

The budget allocation to support affected sectors and economic activity in less-favored areas is a key point for economic growth if these sectors are correctly identified and prices are adjusted according to the specific needs of the area (optimal price transmission mechanism) [13].

The Generally Calculable Balance Model (CGE) [14] develops a theory of quantitative policies for access to socio-economic effects in industries with environmental impact. The result of the model reveals the equation of reproducing the consumption behavior in a controlled economy at a given time. The model details the price transmission mechanism without identifying the influences of dynamic development processes. The model is based on social accounting matrix developed by Czamanski starting to 1970. A new model was realised 27 years later. It starts from the identifying flows between the microeconomic and macroeconomic component through actions of local authority regulations. Finally, the model realises a regional more complex input-output analysis through which disaggregation of regional interrelation multipliers, interregional feedbacks and evaluation of intra-sectoral functional components occur [15].

The social accounting model was applied in order to explain and to quantify the impact of the climate changes on Western Australia. The research covered 71 experiments focused on the winter rainfall and concluded that almost 70% of the observed trend is congruent with the SAM trend in that region. The information in the data series was not modelled stochastically as [16,17,18], but by means of the SAM matrix which revealed the need for new data. On the other hand, the analysis considered that “other forcing factors have to be invoked to fully account for the observed rainfall reduction” [19].

SAM was used in Spain, too. The model was able to estimate the water footprint of Huesca region. The analysis was based on the logical supposition that the water footprint covers the water needed for the production of the goods and services consumed by the inhabitants plus the direct consumption in the households. These consumptions were quantified using an open input-output model. The main result of the analysis was that the agrarian water use represented the greatest water consumer [20]. A similar approach to water resource reduction [21] uses the SAM matrix to simulate the evolution of shadow prices and agricultural output. The authors introduce the following macro indicators into the SAM matrix: macroeconomic variables including GDP, government’s revenues/expenditures, households’ income, and net exports. The results of the study show that the water crisis has a major impact on the agricultural economy, and that the SAM matrix can provide information for sustainable policy formulation.

The use of the SAM matrix in the income distribution analysis was carried out by M. Harun, A. Zakariah, and M. Azali [22]. For this purpose, detailed aggregation and disaggregation of accounts were used which have covered public expenditure in production sectors and household groups. The analysis highlighted the ability of the SAM approach in highlighting chains of linkages from changes in demand to changes in production, factor incomes, household incomes and final demands.

An extension of the SAM matrix in the field of environment was carried out by O. Banerjee, M. Cicowiez, R. Vargas, and M. Horridge [23]. The authors proposed a new model (ESAM–Environmental Social Accounting Matrix), which completed the computerized picture of the interdependent relationship between the economy and the environment in a dynamic, unified and relevant way for issuing social policies on environmental protection.

Another approach to population health during the pandemic [24] was carried out using the SAM matrix. The conclusion of the study shows that based on financial flows it is possible to predict the behaviour of economic agents (social responsibility) in terms of income and expenditure related to combating the effects of the pandemic, including vaccination.

The model was developed later by adding interregional network transfers to a complex theory that presents structured flows that occur within social accounting. The matrix model was also structured and developed by other authors [25,26], who added that they applied for the calculation of sectoral productivity or the calculation of the matrix price multiplier.

The spatial economic interactions and flows patterns under Social Accounting Matrices (SAM) approach led to the conclusion that it can be used to quantify the spatial employment impacts of the Common Agricultural Policy. The SAM was extended, and its structure, assumptions and characteristics were redefined at interregional level [27].

The different methods of building regional SAMs, according to General Equilibrium Models with spatial characteristics were analysed starting from a large literature review. The final goal of this analysis consisted of analysing Colombian regions with such new models [28].

The applicability of SAM was extended to urban development, urbanization projects and urban life cycle forecasting. This is why SAM takes into consideration elements such as the following: heterogeneous land developers, housing consumers and planning agencies. The same SAM can support the solution of dividing the metropolitan areas into a multitude of sprawling cities [29]. 

Czamanski’s approach is used even outside EU27, for improving the management in the enterprises from the food industry in Moldova, for example. The analysis realised a synthesis of the economic concepts in the energetic management from food industry. Moreover, it proposes models and systems of energetic management able to decrease the consumption of energetic resources and to improve the environment [30].

The first intermediate conclusion is that the model further raises the interest of researchers due to the need to correlate macroeconomic policies with flows made at the level of economic entities. As a result, the present analysis proposes a study on the applicability of the regional social accounting matrix on intra-business relations for the top 52 companies in the urban growth area in Galati, Romania.

## 3. Research Methodology

In order to develop the model SAMI, the research started from the analysis of the initial model SAM, which will be transformed according to the research objectives:

O1: Identification of the building vulnerabilities for model SAM according to the present conditions on the global market.
  According to the literature, there are contradictions regarding the mirroring of environmental issues in the SAM matrix [9], correlation of the matrix model with Generally Calculable Balance Model (CGE) [14], or the use of the matrix in connection with the urbanisation processes [29].
O2: Identification of the intrabusiness sectoral flows according to the social accounting criteria.
O3: The literature has taken the first steps in this area [31], highlighting that there is a correlation between intra- and interbusiness connections with implications for consumption, investment and other equilibrium economic relationships.
O4: Defining the social accounting matrix model based on intrabusiness exchanges;
O5: Testing the model for a growth pole in EU27;
O6: Dissemination of the model’s results in connection to the realities in the growth pole;
O7: Evaluating the public policies’ efficiency in the growth pole by using model SAMI.

According to the System of National Accounts, the initial SAM was created to quantify the regional economic transactions. There are differences related to SAM’s translation from national to regional level. As a result, SAM presents the regional economy in a static manner for each year. The static approach represents a challenge for the model, which will be solved in this paper.

SAM represents a classic matrix with its rows and columns which represent the classical economic actors: households, companies, public sector and rest of world. The economic actors are analysed under their double hypostasis: buyers and sellers. As a logical result, the expenditures are quantified on the columns and the revenues from sales are pointed out on the rows.

An important advantage in using SAM matrix is that the number of columns and rows can be increased as the analysis asks [5]. The introduction of the net investment in SAM matrix was realised in 2008 [32]. As a result, the actual SAM matrix structure is that presented in Table 1 [33].

The need of improving SAM matrix started to connect it directly to the regional economic systems [34] and continued to a new approach on Input-Output Analysis [35]. Nowadays, SAM describes the intraregional connections between regional economic actors using the role of different income categories. Moreover, SAM can quantify different regional multipliers as well.

Starting with the matrix model, where consumption represents a direct function of revenues, in relation to a temporal update rate, SAMI (SAMIntrabusiness) matrix proposed in this paper is defined as:

If *c_ij_*–production cost of the good *i* in industry *j* and *q_ij_*–the quantity of goods *i* realised in industry *j* at a given time, then (Ǝ) *C*–a consumption function defined as:

(1) $$C=\sum_{i,j=1}^{n}\left(\gamma_{ij}+\prod_{ij=1}^{n}\left({\left(q_{ij}\right)}^{\left(1-\propto \right)}*{\left(c_{ij}\right)}^{\left(1+\beta \right)}\right)\right),$$

where α, β > 0, α, β, economic multipliers for quantitative and qualitative consumption; $\gamma_{ij}$, the impact of sectoral policies developed by authorities in favor of the business environment; ${\left(q_{ij}\right)}^{\left(1-\propto \right)}$ the quantity of goods *i* realised in industry *j* and purchased by consumers at a certain point in time; and ${\left(c_{ij}\right)}^{\left(1+\beta \right)}$ the sale price of the good *i* in industry *j*. It can be said that *C_j_* is in relation to *R*, according to the next formula:

(2) $$Ri_{\;}=\sum_{i,j=1}^{n}\left(\gamma_{ij}+\prod_{ij=1}^{n}\left({\left(q_{ij}\right)}^{\left(1-\propto \right)}*{\left(c_{ij}\right)}^{\left(1+\beta \right)}\right)-\prod_{ij=1}^{n}\left({\left(q_{ij}\right)}^{\left(\propto \right)}*{\left(c_{ij}\right)}^{\left(1\right)}\right)\right).$$

As a result,$\;C=R-\frac{R}{{\left(1+a\right)}^{t}}$, where *R* is the intrabusiness remuneration. Moreover, *R* represents the update rate in a directly proportional relationship to the impact of sectoral policies developed by authorities in favor of the business environment and *t*–the optimum moment for which the function produces competitiveness. 

Matrix SAMI is defined using the above relations as follows:

(3) $$SAMI=\left[\begin{array}{ccc} \frac{C_{1}}{R_{1}} & \cdots & \frac{C_{1}}{R_{i}}\\ \vdots & \ddots & \vdots \\ \frac{C_{j}}{R_{1}} & \cdots & \frac{C_{j}}{R_{j}} \end{array}\right]\;=\left[\begin{array}{ccc} 1-\frac{1}{{\left(1+a_{1}\right)}^{t_{1}}} & \cdots & 1-\frac{1}{{\left(1+a_{1}\right)}^{t_{i}}}\\ \vdots & \ddots & \vdots \\ 1-\frac{1}{{\left(1+a_{j}\right)}^{t_{1}}} & \cdots & 1-\frac{1}{{\left(1+a_{j}\right)}^{t_{i}}} \end{array}\right]$$

The matrix will focus, under the case study, on inter-business relationships for the 52 companies that are the subject of this study.

## 4. Results and Discussion

As a result of the regional analysis of the economic performance of the top 52 companies in the growth pole Galati, worrying developments regarding turnover in the region were pointed out. The first intermediate conclusion is that the regional decreased by 2.23 million € during the analysed period. 

**Figure 1:**
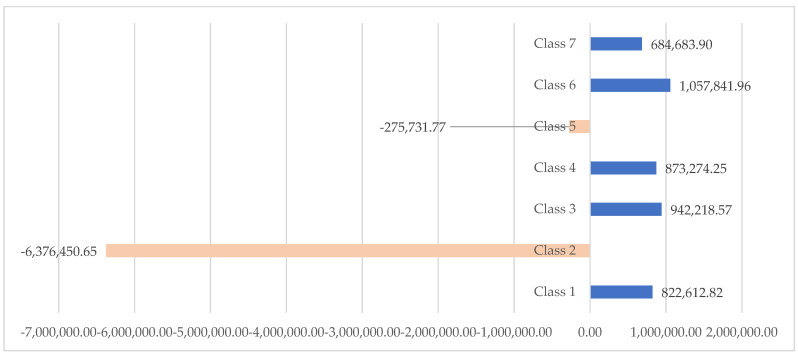
Histogram representation of the distribution of achievements on the Turnover of Top 50 companies in Galati (€). Source: Authors’ processing based on the information provided on Topfirme.com. Accessed date: 5 June 2018.

The main contributor to this decreasing is the metallurgical industry (including steel industry), which achieved an economic counterperformance regarding the turnover of about 6.38 million €, so much that the analysis took into consideration one of the greatest steel factories across the EU27. At the opposite pole, IT services, manufacturing, commerce and construction have brought annual growth in turnover of 0.85–1.06 million €. They have, on average, reduced the negative impact of the increase in turnover in the metallurgical industry (see Figure 1).

The turnover level reveals the structural changes of the regional economy based on the general policy of the local administration, which was more conservative in terms of investment, including infrastructure, while increasing the fiscal pressure on economic agents, notably by increasing taxes. The pollution taxes applied to metallurgical operators were the biggest ones and they were finally materialized in economic branch rebound.

The general trend of profitability faces opposite evolution compared to that of the turnover. The companies from IT services (Class 6) achieved an annual gross profit accumulation of 1.91 million € (1.6 million € net profit). The 16% difference in industry is represented by the taxation of the profit and quantifies direct contributions of micro to the macroeconomic sectors.

The metallurgical industry (Class 2) faces an average profitability of 0.51 million € and no significant difference between gross and net values. This means the withdrawal of companies from the profitability area, the accumulation of losses in the industry and the decrease in productivity. The manufacturing industry (Class 3) achieves the third rank in profitability, with an average annual value of 0.27 million €. A non-performance in terms of profitability is achieved in the transport services segment (Class 5). This industry faces a negative turnover capitalization of 0.28 million € per year and also records annual losses of 0.08 million. €.

**Figure 2:**
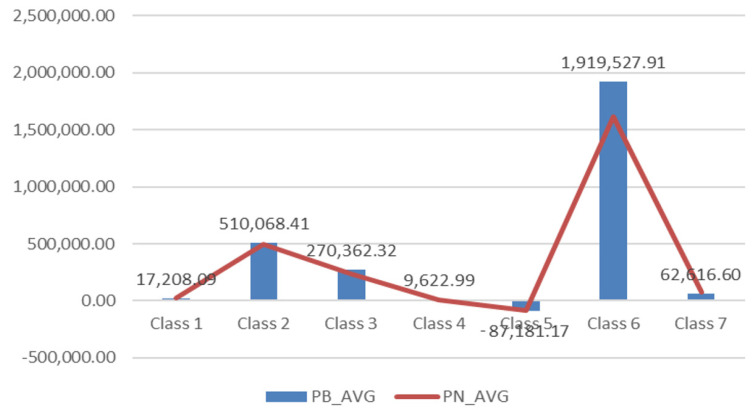
Regional distribution of profitability by industries during 2012–2017. Source: Authors’ processing based on the information provided on Topfirme.com. Accessed date: 5 June 2018.

The losses are motivated by the lack of investment in port infrastructure and the lack of interest of the authorities in the financial support of the branch, which through this measure manages to accumulate a negative trend of the employees as we shall see below (see Figure 2).

**Figure 3:**
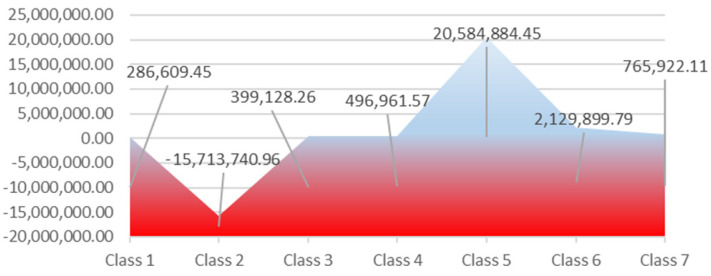
Regional capitalization of the companies at industry level during 2012–2017 (euros). Source: Authors’ processing based on the information provided on Topfirme.com. Accessed date: 5 June 2018.

The company capitalization is positive on average (8.94 million € annual), but the structural differences are significant across the 7 NACE Rew.2 classes (Statistical Classification of Economic Activities in European Community). Thus, the transport services sector proved to be unprofitable and redundant. Under the turnover, the result is an average annual capitalization of 20.64 million €, which means inefficient allocation of resources and inefficient management of social accounting. A second major instability generator is the non-performance of metallurgical industry (Class 2). During 2012–2017, it has recorded an average annual reduction in the capitalization of the five companies of 15.74 million €. As a result, the capital accumulations in the transport services sector decreased. A good performance, but without the support of local authorities–bad management–is recorded by the IT services sector, which achieves an average annual capitalization of 2.13 million €. This capitalization is considered by the authors of this paper as being sustainable in relation to the other mentioned above performances (see Figure 3).

Inventory level is another indicator of bad social management reflecting, for companies in the metallurgical industry, an additional accumulation of 1.43 million €. On the transport services segment, where a capitalization of 20.64 million € was realised annually, the value of stocks increased only by 0.02 million €. Significant inventory accumulations are made in the engineering and technical consulting sector to the capitalization of enterprises (0.74 million € increase in annual inventory versus 0.77 million € increase in capitalization companies). This is unsustainable, as capitalization would be reflected in long-term investment and would be matched by growth in turnover and debt reduction. In this case, the annual debt growth on Class 7–Engineering services is similar to the increase in stocks (0.78 million € compared to 0.74 million € increase in inventories). This points out the need for a different approach to public investment, the need to re-prioritize established objectives and the import of know-how on the social accounting segment.

**Figure 4:**
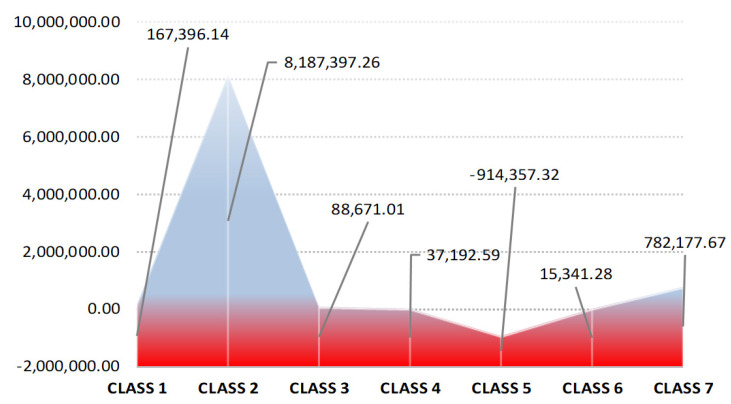
Evolution of average indebtedness at industry level (€). Source: Authors’ processing based on the information provided on Topfirme.com. Accessed date: 5 June 2018.

Low performances in terms of debt growth were recorded in the metallurgical industry, where debt grew by almost 8.19 million € annually (100% of the average annual increase). The other classes, excepting Class 5–Transport services, recorded an increase in debt service of 0.94 million € out of which 0.78 million € related to the engineering and technical consulting services. The transport services (Class 5) recorded a reduction in the debt service by using solutions to support the sector by the public segment (social accounting flows). However, these flows were not effectively managed, and generated bad management, negative profitability and turnover decreasing (see Figure 4).

**Figure 5:**
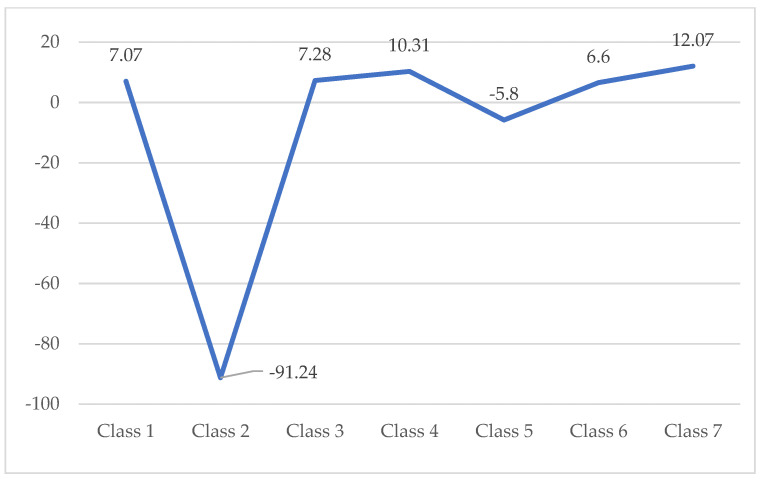
Annual average evolution of the number of employees per industry (persons). Source: Authors’ processing based on the information provided on Topfirme.com. Accessed date: 5 June 2018.

The non-performance of social accounting management can be assessed at first analysis by quantifying the number of employees by activity categories. During 2012–2017, the number of employees decreased by an average of 54 employees, mainly due to the reduction in employees in the metallurgical industry (−91 people annually). In the service, engineering, food, construction and commerce sectors, the annual average growth of employees varied between 12 and 7 employees. This has diminished the negative trend of the metallurgical industry. Even if the capitalization in the transport services is significant, there is a decrease in the number of employees of six persons per year (see Figure 5).

The SAMI matrix at the branch level, calculated on the basis of the moving averages of the period 2012–2017 transposed structured by sectors of activity, in the reference region, is as follows:

(4) $$\begin{array}{cc} SAMI & =\left[\begin{array}{ccc} 1-\frac{1}{{\left(1+a_{1}\right)}^{t_{1}}} & \cdots & 1-\frac{1}{{\left(1+a_{1}\right)}^{t_{i}}}\\ \vdots & \ddots & \vdots \\ 1-\frac{1}{{\left(1+a_{j}\right)}^{t_{1}}} & \cdots & 1-\frac{1}{{\left(1+a_{j}\right)}^{t_{i}}} \end{array}\right]=\left[\begin{array}{ccc} \begin{array}{cc} \begin{array}{c} \begin{array}{c} 2\\ 0\\ 2 \end{array}\\ 2\\ \begin{array}{c} -5\\ 2 \end{array} \end{array} & \begin{array}{c} \begin{array}{c} 0\\ 2\\ 0 \end{array}\\ 0\\ \begin{array}{c} 0\\ 0 \end{array} \end{array}\\ 2 & 0 \end{array} & \begin{array}{c} 0\\ 4\\ \begin{array}{c} -1\\ 0\\ \begin{array}{c} 1\\ -4\\ 2 \end{array} \end{array} \end{array} & \begin{array}{c} -2\\ 0\\ \begin{array}{c} 0\\ 0\\ \begin{array}{c} 0\\ 0\\ -2 \end{array} \end{array} \end{array} \end{array}\right]\underset{i=6;t=7\;}{\Leftrightarrow }\\ & \left[\begin{array}{ccc} \frac{1}{{\left(1+a_{1}\right)}^{t_{1}}} & \cdots & \frac{1}{{\left(1+a_{1}\right)}^{t_{i}}}\\ \vdots & \ddots & \vdots \\ \frac{1}{{\left(1+a_{j}\right)}^{t_{1}}} & \cdots & \frac{1}{{\left(1+a_{j}\right)}^{t_{i}}} \end{array}\right]=\left[\begin{array}{ccc} \begin{array}{cc} \begin{array}{c} \begin{array}{c} -1\\ 1\\ -1 \end{array}\\ -1\\ \begin{array}{c} -4\\ -1 \end{array} \end{array} & \begin{array}{c} \begin{array}{c} 1\\ -1\\ 1 \end{array}\\ 1\\ \begin{array}{c} 1\\ 1 \end{array} \end{array}\\ -1 & 1 \end{array} & \begin{array}{c} 1\\ -3\\ \begin{array}{c} 2\\ 1\\ \begin{array}{c} 0\\ -5\\ -1 \end{array} \end{array} \end{array} & \begin{array}{c} 3\\ 1\\ \begin{array}{c} 1\\ 1\\ \begin{array}{c} 1\\ 1\\ 3 \end{array} \end{array} \end{array} \end{array}\right]\underset{i=6;t=7\;}{\Leftrightarrow }\gamma_{ij}=\left[\begin{array}{ccc} \begin{array}{cc} \begin{array}{c} \begin{array}{c} -1\\ 1\\ -1 \end{array}\\ -1\\ \begin{array}{c} -4\\ -1 \end{array} \end{array} & \begin{array}{c} \begin{array}{c} 1\\ -1\\ 1 \end{array}\\ 1\\ \begin{array}{c} 1\\ 1 \end{array} \end{array}\\ -1 & 1 \end{array} & \begin{array}{c} 1\\ -3\\ \begin{array}{c} 2\\ 1\\ \begin{array}{c} 0\\ -5\\ -1 \end{array} \end{array} \end{array} & \begin{array}{c} 3\\ 1\\ \begin{array}{c} 1\\ 1\\ \begin{array}{c} 1\\ 1\\ 3 \end{array} \end{array} \end{array} \end{array}\right] \end{array}$$

**Figure 6:**
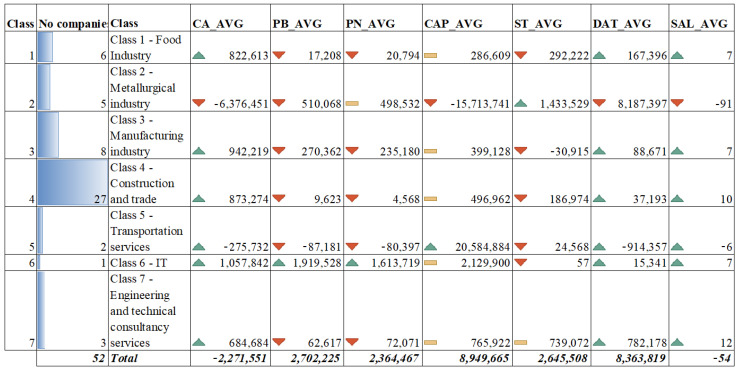
Evolution of performance indicators highlighted by the main categories of financial and patrimonial interest for the studied entities. Source: Authors’ processing based on the information provided on Topfirme.com. Accessed date: 5 June 2018.

The data presented as a matrix flow are derived from the calculation of the moving averages over the 6 years of the study (2012–2017) for the 52 companies grouped in seven areas of interest, of which the metallurgical sector is by far the most unsatisfactory. In dynamics, the data structure is presented according to the data in Figure 6. The following notes and formulas have been used:

-CA_AVG represents the average dynamic industry turnover between 2012 and 2017 and is calculated according to the formula:

(5) $$A\_\mathrm{AVG}=\frac{\sum_{i=1}^{7}\sum_{t=1}^{6}\left(CA_{it}-CA_{it-1}\right)*n_{it}}{\sum_{t=1}^{6}n_{it}};$$

-PB_AVG represents the average dynamic industry gross profit between 2012 and 2017 and is calculated according to the formula:

(6) $$\mathrm{PB}\_\mathrm{AV}=\frac{\sum_{i=1}^{7}\sum_{t=1}^{6}\left(PB_{it}-PB_{it-1}\right)*n_{it}}{\sum_{t=1}^{6}n_{it}}$$

-PN_AVG represents the average dynamic industry net profit between 2012 and 2017 and is calculated according to the formula:

(7) $$\mathrm{PN}\_\mathrm{AVG}=\frac{\sum_{i=1}^{7}\sum_{t=1}^{6}\left(PN_{it}-PN_{it-1}\right)*n_{it}}{\sum_{t=1}^{6}n_{it}}$$

-CAP_AVG represents the average dynamic capitalization of companies by industry during 2012–2017 and is calculated according to the formula:

(8) $$\mathrm{CAP}\_\mathrm{AVG}=\frac{\sum_{i=1}^{7}\sum_{t=1}^{6}\left(CAP_{it}-CAP_{it-1}\right)*n_{it}}{\sum_{t=1}^{6}n_{it}}$$

-ST_AVG represents the average stock accumulation by industries in dynamics during 2012–2017 and is calculated according to the formula:

(9) $$\mathrm{ST}\_\mathrm{AVG}=\frac{\sum_{i=1}^{7}\sum_{t=1}^{6}\left(ST_{it}-ST_{it-1}\right)*n_{it}}{\sum_{t=1}^{6}n_{it}}$$

-DAT_AVG represents the average dynamic debt ratios per industry between 2012 and 2017 and is calculated according to the formula:

(10) $$\mathrm{DAT}\_\mathrm{AVG}=\frac{\sum_{i=1}^{7}\sum_{t=1}^{6}\left(DAT_{it}-DAT_{it-1}\right)*n_{it}}{\sum_{t=1}^{6}n_{it}}$$

-SAL_AVG represents the average of labor dynamic absorption by industries between 2012 and 2017 and is calculated according to the formula:

(11) $$\mathrm{SAL}\_\mathrm{AVG}=\frac{\sum_{i=1}^{7}\sum_{t=1}^{6}\left(SAL_{it}-SAL_{it-1}\right)*n_{it}}{\sum_{t=1}^{6}n_{it}}$$

The classes presented in Figure 6 are defined as follows:

(a) Class 1–Food industry;
(b) Class 2–Metallurgical industry;
(c) Class 3–Manufacturing industry
(d) Class 4–Construction and trade;
(e) Class 5–Transport services;
(f) Class 6–IT services;
(g) Class 7–Engineering and technical consultancy services.

The above presented data are interpreted according to impact indicator allocation keys (symbol of change in importance according to Figure 6). These data, through comparative study, can be structured in five major categories of socio-accounting influence, namely:

(a) Lack of efficiency or lack of public policies;
(b) Fiscal requirements;
(c) Lack of market, inefficiency of intra-business exchanges;
(d) Increasing indebtedness;
(e) Effectiveness of social policies.

This structure has highlighted some areas that need to be monitored and some areas that need to be thoroughly revised in order to reduce impact and mitigate effects.

The trends of the indicators presented in Figure 6 have been quantified as follows:

(a) Quote 1–upward trend over the average growth rate, coefficient 5;
(b) Quote 2–upward trend as the average of the indicator, coefficient 3;
(c) Quote 3–rising trend below the average growth rate of the indicator, coefficient 1;
(d) Quote 4–downward trend of the indicator, coefficient 0.

In order to quantify the effectiveness of public policies, the evolution trends of the indicators CA_AVG and CAP_AVG, coded according to quotes 1 to 4 above, have been compared. The result consists of a difference in the efficiency of public policies by classes of activity (see Table 2).

In order to quantify the effectiveness of the fiscal policies, the evolution trends of the indicators PB_AVG și PN_AVG, coded according to quotes 1 to 4 above have compared. The result consists of a difference in the efficiency of fiscal policies by classes of activity (see Table 3).

The next step was to quantify the effectiveness of the intra-business exchanges, the evolution trends of the indicators ST_AVG and PB_AVG, coded according to quotes 1 to 4 above have been compared. The result consists of a difference in the efficiency of intrabusiness exchanges by classes of activity (see Table 4).

The quantifying of the microeconomic remuneration efficiency in relation to increasing indebtedness implies the analysis of the evolution trends of the DAT_AVG and CAP_AVG indicators, coded according to above quotes 1 to 4, resulting in a difference in the efficiency of microeconomic remuneration by classes of activity (see Table 5).

Finally, in order to quantify the social policies efficiency, the analysis took into consideration the evolution trends of the SAL_AVG and CA_AVG indicators, coded according to above quotes 1 to 4, resulting in the lack of efficiency of social policies in the reference area–the growth pole l Galati, Romania (see Table 6).

**Figure 7:**
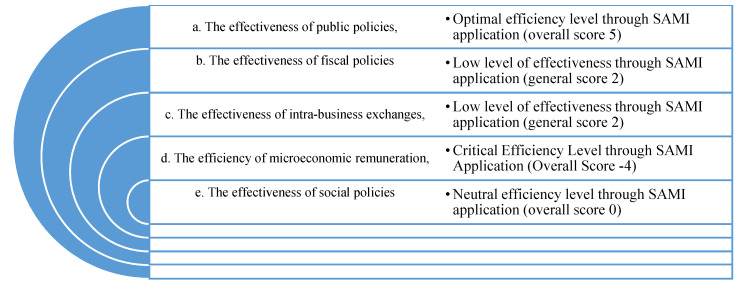
SAMI’s scheme developed by the authors.

The above indicators were computed by applying the impact coefficients to the annual trend of the indicator for the six categories of the SAMI matrix. The significance values of the efficiency level are shown in Figure 7:

The model applied at the inter-business level brings real benefits to all actors involved in regional trade exchanges by assessing the regional level of social accounting development and by highlighting the critical points through which the system fails due to bureaucratic or bad management policies.

To achieve Objective 1 of the research (O1: Identification of SAM model building vulnerabilities in relation to the current global market conditions), a critical analysis of the vulnerabilities of the SAM model was developed based on the literature review.

The regional accounting model was updated 25 years later [15]. This was the result of the model inability to satisfy the quantifying of the regional development. As a result, the new one can measure the long-term potential growth of a regional economy, while the analysis takes into consideration the changes in invested capital, human capital, natural resources, infrastructure and environmental factors. The resulting SAM matrix points out the main goods and monetary flows (see Table 7).

The authors of the paper succeeded in improving the meaning of the approach in the above figure. On the other hand, the model is based on the classic SAM matrix.

A new intermediate conclusion is that Czamanski is not able to escape from the input-output prison’s approach. This is why he continued to use the linear interdependencies between the industries, economic sectors and economic actors.

There are some challenges related to welfare, as well. From point of view of the authors of this paper, welfare is not dimensioned only by goods and services. Of course, it depends directly on the income level, but it does not cover only consumption, income and higher savings. There are enough elements which cannot be bought with money and have contributions to welfare. As a result, income is able only to approximate the individuals’ and other economic actors’ welfare.

An interesting approach is that money is able to contribute indirectly to greater happiness [36]. The economic realities presents many situations when the aggregate income’s increase doesn’t have correspondent in individuals’ welfare (The increase in the duration and intensity of work done by each individual or the increase in the retirement age bring more money but not more wealth).

There are other outputs (weapons for example) which have no effect on the individuals’ welfare even that they contribute to an increase in income (GDP). The lower quality outputs have the same effect. These two examples lead to the conclusion that welfare is directly related to consumption, not to production [37].

If the increase in the average and aggregate income is doubled by an unfair distribution of income in two countries which have the same average income, the effects on welfare vary a lot. A relatively similar effect comes from the government policy differences in income distribution and redistribution.

A limit of SAM approach is that it does not take into consideration the underground economy and the fact that the output increasing is not always equal to the consumption increasing. Moreover, the SAM matrix takes into consideration the externalities only under the regional endowment.

All deficiencies previously identified have been reviewed and resolved within the proposed SAMI model. At this moment, the goals of the Objective 1 were satisfied. 

The present research responds to the proposed Objective 2 (O2: Identify intrabusiness sectoral flows according to social accounting criteria) by calculating the efficiency of intra-business exchanges (see Table 3). The efficiency scoreboard was built on activity classes and allowed comparative analysis of sustainable accumulation in terms of profitability as a result of inventory dynamics by classes of activity over the period 2012–2017, the period for which full data were reported for the 52 companies included in the sample. This is because some of the companies have barely entered in the top in recent years.

The matrix model proposed in this paper was defined in the methodology section. As a result, the proposed Objective 3 (O3: Defining the social accounting matrix model based on intrabusimess exchanges) was also met.

The testing of the proposed model was carried out in steps to assess the effectiveness of public policies, fiscal policies, intra-business exchanges, assessing the efficiency of microeconomic remuneration and the effectiveness of social policies. These tests have allowed the construction of NACE Rev.2 and have demonstrated the sustainability of the model by obtaining valid results, micro, macro and regional causality correlations. As a result, the Objective 4 of the research (O4: Testing the growth model for the EU27) has been validated.

The results were disseminated and a general regional efficiency table for the growth pole was built (see Table 5). The Objective 5 of the research was also achieved (O5: Dissemination of model results in relation to market realities in the growth pole).

The final objective, Objective 6, of the research reveals the vulnerability of the public policies promoted in the growth pole (O6: Evaluation of the efficiency of public policies in the growth pole by applying the SAMI model), making a precise radiography of the government’s bad management with a direct impact on the bad management of the economic agents.

The main regional deficiencies concern the poor quality of the transport infrastructure (lack of airport, lack of interconnection with the TEN-T network, administrative bureaucracy, inefficiency in the management of the free zone), factors that inhibit sustainable development in the region benefiting from the existence of the river transport infrastructure, the existence of economic agents as locomotives of the economic growth (DAMEN, ARCELOR, Mineral Port), the existence of a university center of tradition, etc.

The results of the study reflect the fact that the SAMI matrix is a forward-looking tool that addresses the shortcomings identified in Table 7 and systematizes the linkages between businesses conducted regionally for representative firms (with regional decision-making power). 

The aggregate distribution function reflects the fact that relevant models can be designed based on the systematization of the decision within the SAMI matrix, eliminating vulnerabilities through distributions and redistributions leading to sustainable policy making. 

The objectives achieved in this research may constitute new working hypotheses for future extended research in Ecology and environment.

## 5. Conclusions and Policy Implications

Through this research, the authors proposed the development of the SAMI matrix for intra-business relations. The study has a high practical applicability through easy-to-use methodology, the punctual diagnosis of intra-business flows and by identifying the vulnerabilities of the social accounting system.

The results of the study can be applied both by the representatives of the local authorities as well as by the top management and the stakeholders of economic units generating economic growth.

There is no doubt that the present approach has a strong connection to the public policies’ implementation. For the beginning, this approach is based on main categories of financial and patrimonial interest for the studied entities. All these entities are elements of the public policies for sustainable development.

On the other hand, the public policies have to be efficient. As a result, the authors succeeded in quantifying the effectiveness of public policies using seven classes of activity, two indicators (CA_AVG and CAP_AVG) and dedicated values (from 0 to 5). According to these, the analysis was able to identify the Transport services as an inefficient industry which has to be a priority for the public policy (see Table 2).

A distinct analysis covers the fiscal policies. The result of this analysis is that these policies had a good impact on the Metallurgical industry (see Table 3).

Moreover, the authors of this paper proposed a new instrument (SAMI) which is able to assist the public policy decision makers in improving the implementation of specific public policies and in obtaining better performances. These performances cover the classical four economic actors (business, households, governments and rest of the world) but take into consideration the intergenerational transfers regarding Investments and Regional endowment. 

The final goal of SAMI is the analysis of intra-business exchanges, quantification of financial flows and strategic development agreements in the long-term for economic entities through global partnerships (see Table 7). All of these are elements of the public policies, as well.

We can conclude that the proposed model proved to be valid by correlating the empirical demonstration with the values resulting from the applicative study, the identified problems being real and very current.

The limitations of the study consist of the small number of indicators taken into account and the relatively limited scope of data analysis, the authors proposing to deepen their current study in a future article by refining input use and broadly defining financial outputs and the ecology and environment under the impact of the COVID-19 pandemic.

## Figures and Tables

**Table 1:** Scheme of SAM. Source [33].

	Companies	Households	Public Sector	Other	Net Investment	Total (Revenues)
Companies		C	G_F_	(X − I)_K_	Inv	C + G_F_ + (X − I)_K_ + I
Households	W		G_H_	(X − I)_C_		S + G_H_ + (X − I)_C_
Public sector	T_F_	T_H_				T_F_ + T_H_
Other	(X − I)_K_	(X − I)_C_				(X − I)_K_ + (X − I)_C_
Net investment		S_H_	S_G_			S_H_ + S_G_
Total (expenditures)	S + T_F_ + (X − I)_K_	C + T_H_ + (X − I)_C_ + S_H_	G_F_ + G_H_ + S_G_	(X − I)_C_ + (X − I)_K_	Inv	

Where: T–taxes; S–wages; I–imports; E–exports; S–savings; Inv–investment; C–consumption; G–governmental sector. Indexes: F–firms; H–households; G–governmental sector; C–goods consumption; K–capital.

**Table 2:** Evaluating the effectiveness of public policies.

Class of Activity	CA_AVG	CAP_AVG	Effectiveness of Public Policies	Effectiveness Level
Class 1–Food industry;	5	3	2	Efficient
Class 2–Metallurgical industry;	0	0	0	Neutral
Class 3–Manufacturing industry	5	3	2	Efficient
Class 4–Construction and trade;	5	3	2	Efficient
Class 5–Transport services;	0	5	−5	Inefficient
Class 6–IT services;	5	3	2	Efficient
Class 7–Engineering and technical consultancy services.	5	3	2	Efficient

Red: Inefficient; Yellow: Efficient.

**Table 3:** Evaluating the effectiveness of fiscal policies.

Class of Activity	PB_AVG	PN_AVG	Effectiveness of Fiscal Policies	Effectiveness Level
Class 1–Food industry;	1	1	0	Neutral
Class 2–Metallurgical industry;	1	3	2	Efficient
Class 3–Manufacturing industry;	1	1	0	Neutral
Class 4–Construction and trade;	1	1	0	Neutral
Class 5–Transport services;	0	0	0	Neutral
Class 6–IT services;	5	5	0	Neutral
Class 7–Engineering and technical consultancy services.	1	1	0	Neutral

Yellow: Efficient.

**Table 4:** Assessing the effectiveness of intra-business exchanges on sales markets by classes of activity.

Class of Activity	ST_AVG	PB_AVG	Effectiveness of Intra-Business Exchanges	Effectiveness Level
Class 1–Food industry;	1	1	0	Neutral
Class 2–Metallurgical industry;	5	1	4	Maximum Efficient
Class 3–Manufacturing industry;	0	1	−1	Inefficient
Class 4–Construction and trade;	1	1	0	Neutral
Class 5–Transport services;	1	0	1	Efficient
Class 6–IT services;	1	5	−4	Inefficient
Class 7–Engineering and technical consultancy services.	3	1	2	Efficient

Red: Inefficient; Green: Maximum Efficient; Yellow: Efficient.

**Table 5:** Evaluation of the efficiency of the microeconomic remuneration in relation to the increase in the indebtedness by classes of activity.

Class of Activity	DAT_AVG	CAP_AVG	Efficiency of the Microeconomic Remuneration	Efficiency Level
Class 1–Food industry;	1	3	−2	Inefficient
Class 2–Metallurgical industry;	0	0	0	Neutral
Class 3–Manufacturing industry;	3	3	0	Neutral
Class 4–Construction and trade;	3	3	0	Neutral
Class 5–Transport services;	5	5	0	Neutral
Class 6–IT services;	3	3	0	Neutral
Class 7–Engineering and technical consultancy services.	1	3	−2	Inefficient

Red: Inefficient.

**Table 6:** Evaluating the effectiveness of social policies.

Class of Activity	SAL_AVG	CA_AVG	Efficiency of the Social Policies	Efficiency Level
Class 1–Food industry;	5	5	0	Neutral
Class 2–Metallurgical industry;	0	0	0	Neutral
Class 3–Manufacturing industry;	5	5	0	Neutral
Class 4–Construction and trade;	5	5	0	Neutral
Class 5–Transport services;	0	0	0	Neutral
Class 6–IT services;	5	5	0	Neutral
Class 7–Engineering and technical consultancy services.	5	5	0	Neutral

**Table 7:** SAM vs. SAMI analysis.

Indicators	Business	Households	Governments	Rest of the World	Intergenerational Transfers		Output
					Investments	Regional Endowment	
Business	Intermediate goods and services	Personal consumption	Current governmental purchases of goods and services	Net exports; Dividends and interest from rest of the world	Net domestic private investments; Public investments.	Technical progress; Depletion of non-renewable resources, renewable resources, soil resources; Degradation of the environment	*Adjusted net regional/national product (NRP)*
SAM results	International analysis without globalisation factor	Traditional consumption evaluation under the influence of international markets	Analysis of government policies in the context of the international markets’ expansion	Trade agreements in the context of customs protectionism	National Strategic Investment Policies	Technical Progress analyzed from the perspective of competitive advantage	Business services aiming at territorial expansion and winning international markets
SAMI results	Strength model, including globalisation factor; Business to business analysis.	Evaluation of the global acquisition for the digitized society under the influence of the global markets	Analysis of Cohesion Policies in context of the global markets	Multinational corporative policies in the context of free movement of goods and services	The role of FDI in the context of globalization; Financing schemes at European level	Technical Progress in the global digitized society	Analysis of intra-business exchanges, quantification of financial flows and strategic development agreements on long-term for economic entities through global partnerships
Households	Wages, salaries, other labour income; Net personal interest; Proprietor’s income; Rental income; Business transfers		Net interest paid by governments; Transfer payments less social security contributions			Increase in human capital	
SAM results	Labour productivity analysis by intruder Input- Output method		Assessing the success of social policies through their monetary side	SAM model does not quantify the elements of labor migration and the global change in consumer behavior under the impact of global trade	SAM model doesn’t cover intergenera-tional transfers related to investments	SAM evaluates inter-regional transfers at the household level by increasing the value of the skilled labour.	The outputs of the SAM matrix are limited to the development of human capital in the traditional context of national markets engaged in international competitions.
SAMI results	Introduction of CSR values and staff motivation for optimizing labor productivity		Quantifying sustainable social protection efforts through social cohesion policies at European level	The elements of global trade are quantified by intrabusiness financial flows	SAMI, through its regional component, quantifies the impact of investments on households	Aspects regarding labor migration and social protection of European citizens through CBM community monitoring mechanisms are introduced in the SAMI matrix	SAMI integrates innovative concepts on social responsibility and community monitoring to assess the impact of households in social accounting
Govern-ments	Corporate taxes; Social security taxes; Indirect taxes; Surplus of government enterprises less subsidies	Personal taxes; Local taxes				Increase in value of subsoil resources	
SAM results	Financing the economy through the business component is quantified through SAM exclusively through taxation.	Financing the economy through the social component is quantified through SAM exclusively through taxation.		SAM does not assess the financing of macroeconomics through external financing mechanisms (European Sector Financing Scheme, for example).	SAM does not quantify the intra-regional impact of the investments.	SAM sets the foundations for sustainable development concepts but does not assess them at the current market demands’ level.	SAM carries out a fiscal quantification of the macroeconomics’ remuneration through the classic components of fiscal mechanisms and does not correspond to the current requirements on the complexity of fiscal policies applied at regional level.
SAMI results	The introduction of intra-business flows strengthens the financing component by adding the multiplier effect of the economy to the commercial and investment chains.	SAMI highlights regional labor transfers as a potential development factor.		SAMI encompasses European funding schemes in the context of macro and micro financial flows.	SAMI quantifies the intra-regional impact of the investments.	SAMI quantifies the sustainability objectives mentioned in Agenda 2030.	SAMI quantifies intra-business exchanges and sustainable development components. SAMI improves the prospect of macroeconomic remuneration on the society’s integrated financial flows based on interchange.
Rest of the world			Transfer payments		Net investment or disinvestment in the rest of the world		
SAM results	SAM does not quantify global intrabusiness flows.	Phenomena such as migration of qualified labour with impact on the development of a region (concrete case analyzed in the paper) are not analyzed by SAM. The impact of labour transfer through the size of goodwill elements at regional entity level is a major vulnerability of the model.	The monetary payment system, as a support to the national economy, is addressed by SAM in payments balances’ equilibrium, but the global dimensions to which national monetary systems must currently face cannot be quantified.		SAM quantifies net investment as growth support. Currently, under the pressure of multinationals, the SAM model does not distinguish between national private and public net investments.	SAM does not quantify this component.	The international component is partially quantified by SAM by taking into account the transfer of payments and net investment.
SAMI results	SAMI quantifies global intrabusiness flows.	SAMI quantifies goodwill by identifying intra-business flows.	The efficiency of public policies quantified by the model translates precisely the need for funding based on the global trade of national economies, including up to the regional level–growth poles.		SAMI quantifies net investment as growth support. Currently, under the pressure of multinationals, the SAMI model distinguishes between private and public national net investments.	SAMI is focused on regional development, intra-business flows and the expansion of public-private entropy in the region.	The international component is quantified by SAMI at regional level by increasing system entropy and highlighting globalization in all its aspects.
Savings	Retained earnings NRP	Personal savings	Governments’ surplus or deficit on current account				
SAM results	SAM quantifies performance through the Adjusted net regional/national product (NRP), calculating a global accumulation value based on Input–Output method.	SAM quantifies indirect welfare through personal savings.	SAM quantifies social welfare through the surplus or deficit of the balance of payments.	SAM does not quantify the impact of global trade on the region’s welfare.		SAM doesn’t quantify the regional dimension of welfare.	Welfare is calculated by extrinsic accumulation function.
SAMI results	SAMI quantifies the transaction performance and economic chain which gives it greater sustainability and greater entropy of the analyzed regional system.	SAMI takes into account the social policy elements introduced by international cohesion agreements and precisely quantifies welfare based on the capacity of the system to provide long-term sustainable growth.	SAMI quantifies the viability of the macro system through demand-supply interaction with the economic environment in the region, which highlights the entropy of the system.	SAMI quantifies the impact of global trade on the regional welfare.		SAMI accomplishes this by introducing into the equation the assessment of sustainable regional development.	SAMI quantifies welfare through sustainable accumulation as well as through intra-business flows.
Changes in wealth					Net increase in assets	Net increase in endowment	Net increase in wealth
SAM results	SAM does not quantify the asymptotic curves of the evolution of welfare at the level of economic agents.	SAM does not quantify changes in population welfare, this being a vulnerability of the system.	The national economic performance materialized in the medium- and long-term growth of the economy is omitted by the SAM model.	The impact of global trade on economic well-being is ignored by SAM, which is destabilized in fulfilling its function of assessing social accounting as a whole.	SAM evaluates intra-regional investment transfers through net asset growth, not highlighting the qualitative side induced by technological progress.	SAM performs a rough assessment of the endowment growth with an emphasis on environmental components, making a preliminary review of sustainable development.	SAM concretises the change of welfare by conceptualizing net growth in welfare, which represents the quintessence of the model.
SAMI results	SAMI aims at calculating the asymptotic curves for NACE Rev2 activity domains evaluating on their basis the interaction of economic policies with the business environment (see Figure 7).	SAMI, through regional dimension and intrabusiness flows quantification, places individuals as beneficiaries of the system and evaluates population welfare based on sustainable regional development.	These changes include macro phenomena such as inflation/deflation, unemployment, population migration, etc. and are quantified and evaluated at the level of public policy efficiency through the proposed SAMI model.	SAMI involves assessing the impact of global trade on regional sustainable development for growth poles and materializes in a multimodal complex of social accounting.	SAMI focuses on competitiveness and relates intra-regional transfers both to technological progress and to know-how.	SAMI evaluates all aspects of sustainable regional development, and through intrabusiness flow components highlights the effectiveness of sustainable development in growth poles.	SAMI emphasizes net growth in welfare through sustainable policy components and intra-business flows, potentiating their application to current market conditions.

Source: Authors’ contribution.

## Data Availability

We used public data from Eurostat and the World Health Organization.
